# Expression, purification and application of a recombinant, membrane permeating version of the light chain of botulinum toxin B

**DOI:** 10.1042/BSR20240117

**Published:** 2024-07-30

**Authors:** Micaela Vanina Buzzatto, Fabiana Cristina Benegas Guerrero, Pablo Ariel Álvarez, María Paz Zizzias, Luis Mariano Polo, Claudia Nora Tomes

**Affiliations:** 1 Instituto de Histología y Embriología de Mendoza (IHEM)-CONICET-Universidad Nacional de Cuyo, Argentina; 2 Facultad de Ciencias Exactas y Naturales, Universidad Nacional de Cuyo, Argentina

**Keywords:** botulinum toxin B, cell penetrating, exocytosis, sperm cells, synaptobrevin

## Abstract

Botulinum neurotoxins (BoNTs) are valuable tools to unveil molecular mechanisms of exocytosis in neuronal and non-neuronal cells due to their peptidase activity on exocytic isoforms of SNARE proteins. They are produced by *Clostridia* as single-chain polypeptides that are proteolytically cleaved into light, catalytic domains covalently linked via disulfide bonds to heavy, targeting domains. This format of two subunits linked by disulfide bonds is required for the full neurotoxicity of BoNTs. We have generated a recombinant version of BoNT/B that consists of the light chain of the toxin fused to the protein transduction domain of the human immunodeficiency virus-1 (TAT peptide) and a hexahistidine tag. His_6_-TAT-BoNT/B-LC, expressed in *Escherichia coli* and purified by affinity chromatography, penetrated membranes and exhibited strong enzymatic activity, as evidenced by cleavage of the SNARE synaptobrevin from rat brain synaptosomes and human sperm cells. Proteolytic attack of synaptobrevin hindered exocytosis triggered by a calcium ionophore in the latter. The novel tool reported herein disrupts the function of a SNARE protein within minutes in cells that may or may not express the receptors for the BoNT/B heavy chain, and without the need for transient transfection or permeabilization.

## Introduction

Botulinum toxins (BoNTs, serotypes A-G) are produced by anaerobic, spore-forming, Gram-positive bacilli *Clostridia*. Toxins are produced as 150 kDa single-chain polypeptides proteolytically activated into a heavy (100 kDa) and light (LC, 50 kDa) chains that remain connected via disulfide bridges. The heavy chains consist of two structurally independent portions: a receptor-binding domain, responsible for the interaction with membrane receptors on cholinergic neurons and endocytosis of the complex, and a translocation domain, which delivers the LC from the endosomal compartments into the cytosol. The LCs are Zn^2+^-dependent endopeptidases that specifically cleave peptide bonds in one or more soluble N-Ethylmaleimide-sensitive factor attachment receptor (SNARE) proteins. These proteins are not active as single molecules. However, SNAREs residing in the two membranes destined to fuse and assembled in *trans* complexes are at the core of the nanomachines that mediate the docking and fusion of exocytotic vesicles and the plasma membrane [1,2]. Because proteases attack predominantly unstructured exposed loops, only the non-complexed SNAREs can be cleaved by toxins [3]. Proteolytic cleavage of the neuronal isoforms of SNARE proteins by neurotoxins potently inhibits secretory vesicle release [4–6] because cleaved SNAREs are unable to engage in stable complexes [3]. In the research laboratory, toxins provided convincing evidence toward the requirement of SNAREs for exocytosis in several cell types.

At cholinergic nerve terminals, blocking the SNARE complex assembly causes inhibition of neurotransmitters’ release and leads to the potentially fatal flaccid paralysis characteristic of botulism [7–9]. On the other hand, the exceptional biological properties of BoNT, including exquisite neuroselectivity, specific enzymatic activity, and limited diffusion from the injection site have been exploited for beneficial purposes. BoNT/A and B are extensively used as therapeutic and cosmetic agents in the clinic. Their principal uses are in the treatment of disorders involving the hyperactivity of cholinergic fibers innervating skeletal muscles and in the temporary smoothing of facial wrinkles. It has recently become clear that BoNTs have a much wider range of applications than appreciated initially; hence, novel indications have emerged in many fields [6].

As holotoxins exhibit extreme toxicity (they are the most potent poisons for mammals), several strategies have been developed to avoid the risk of handling them such as: reconstitution of functional BoNTs from individual subunits expressed in heterologous hosts [9,10]; transfection with plasmids encoding BoNTs LCs for transient expression in cultured cells; delivery through patch clamp pipettes of recombinant LC; and exposure of cells with their plasma membrane permeabilized with detergents or pore-forming toxins to recombinant LCs.

There are many advantages to using recombinant techniques to express BoNTs heterologously in *Escherichia coli*, a more tractable bacterium than *Clostridium*. It is also safer to express, purify and manipulate LCs than holotoxins. We were interested in generating a tool applicable in short timeframes and suitable to unveil SNARE-dependent pathways in live cells. Here, we describe a biologically active recombinant version of the LC of BoNT/B fused to the cell-penetrating peptide TAT from the immunodeficiency virus-1. The cDNA encoding BoNT/B-LC was inserted in a pET28a (+) expression vector, with the sequences encoding His_6_ tag and TAT upstream its 5′ end. This tool increases the repertoire of membrane-penetrating recombinant neurotoxins, consisting of TAT-BoNT/A-LC-His_6_, whose target is the SNARE SNAP-25 [11] and His_6_-tetanus toxin (TeTx)-LC, whose target is synaptobrevin [12]. His_6_-TAT-BoNT/B-LC, but not His_6_-BoNT/B-LC, rapidly crossed intact membranes. Furthermore, His_6_-TAT-BoNT/B-LC cleaved synaptobrevin in the two model systems tested herein and inhibited the exocytosis (acrosome reaction, AR) elicited by a calcium ionophore in human sperm cells, resembling what His_6_-BoNT/B-LC does in permeabilized cells. Thus, this engineered BoNT shows application potential in the preliminary proof of concept study described in this manuscript.

## Methods

### Reagents

The mouse monoclonals anti-synaptobrevin-2 antibody (clone 69.1, purified IgG) and anti-syntaxin 1A and B (clone 78.2, ascites) were from Synaptic Systems (Göttingen, Germany). The mouse monoclonal anti-His_6_ from Clontech Laboratories, Inc. (a Takara Bio Company) and HisTrap™ columns, FF, Cytiva (formerly GE HealthCare Life Sciences) were purchased from ALLSCIENCE, LLC (Doral, FL). FITC-coupled *Pisum sativum* agglutinin (FITC-PSA) was from EY (San Mateo, CA). Horseradish peroxidase- and Cy3^™^-conjugated goat anti-mouse IgGs (H+L) were from Jackson ImmunoResearch (West Grove, PA). Human Tubal Fluid (HTF) media was from Serendipia Lab (Vedia, Argentina). Ni-NTA-agarose was from GE HealthCare. Prestained molecular weight markers were from Bio-Rad (Tecnolab). All other chemicals were from Sigma-Aldrich™ Argentina S.A., Genbiotech, One Lab or Tecnolab (Buenos Aires, Argentina).

### Construction, expression and purification of His_6_-TAT-BoNT/B-LC

**Figure 1:**
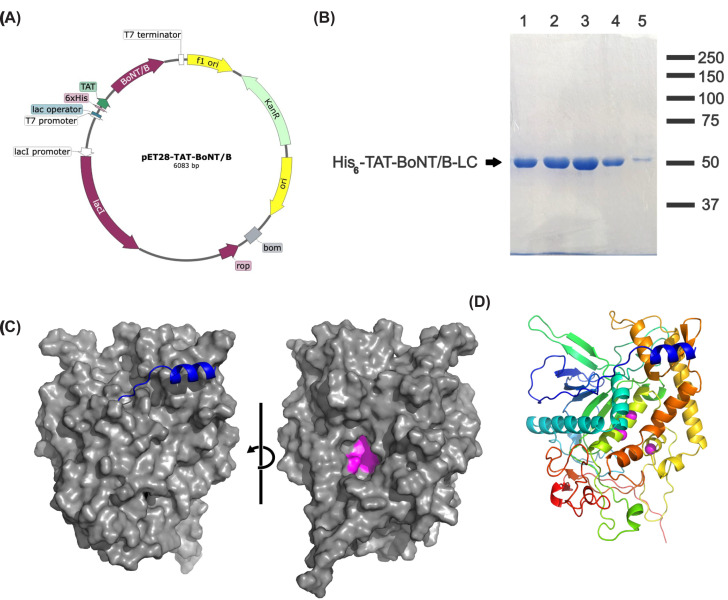
Designed His_6_-TAT-BoNT/B-LC (**A**) Schematic view of designed His_6_-TAT-BoNT/B-LC construct on pET28 expression vector. (**B**) SDS-PAGE stained with Coomassie Brilliant Blue R250. Lanes 1–4 show enriched eluates (buffer containing 250 mM imidazole) from a batch purification. *Mr* standards (×10^3^ Da) are indicated on the right. (**C**) Two orthogonal views, rotated 180°, revealing the solvent-accessible surface of the BoNT/B-LC domain in grey, with the His_6_-TAT peptide depicted as a blue cartoon. This representation emphasizes that the N-terminal tag and the BoNT/B-LC active site (coloured in pink) are positioned on opposite sides of the structure. The representation corresponds to that predicted by RoseTTAfold. (**D**) Secondary structure cartoon representation of His_6_-TAT-BoNT/B-LC predicted by RoseTTAfold, showcasing the folding of the His_6_-TAT tag as a blue alpha helix over the toxin. The molecular cartoon is color-coded from blue to red, representing the N to C-terminus progression.

The cDNA encoding a membrane penetrating version of BoNT/B-LC optimized for bacterial expression was synthesized by Genscript (Piscataway, NJ). The insert was subcloned in frame with the TAT encoding sequence (RKKRRQRRR) of HIV virus for protein transduction into the HindIII-BamHI cloning site of pET28a(+) vector (Novagen, now Merck-Millipore). The cDNA encoding His_6_-TAT-BoNT/B-LC (Figure 1A, nucleotide sequence in Supplementary Material S1) was transformed into *E. coli* BL21(DE3) T1^R^. Selected transformants were inoculated into 5 ml of LB broth containing 50 μg/ml kanamycin and grown under three experimental conditions to optimize protein expression: 0.1 mM isopropyl-β-D-thio-galactoside (IPTG), overnight, 22°C, 200 rpm; 0.5 mM IPTG, 3 h, 37°C, 200 rpm; and 1 mM IPTG, 3 h, 37°C, 200 rpm. Affinity purification of the His_6_-TAT-BoNT/B-LC from these small cultures was carried out under native conditions on Ni-NTA-agarose beads in batch, following the protocol listed in The QIA*expressionist*™ manual (Qiagen GmbH, Hilden, Germany). All fractions were subjected to 10% (w/v), Tris-glycine, sodium dodecyl sulfate-polyacrylamide gel electrophoresis (SDS-PAGE) and resolved proteins were stained with Coomassie Brilliant Blue R250. The condition 0.1 mM IPTG, overnight, 22°C was selected to scale up to larger cultures because it provided the highest yield and purity.

Pellets from 250 ml cultures were suspended in lysis buffer (50 mM NaH_2_PO_4_, pH 6.2, 300 mM NaCl, and 10 mM imidazole), sonicated on ice 10 s at 20 Hertz, added 1% (v/v) Triton X-100 plus a cocktail of protease inhibitors (Sigma) and sonicated twice as before. After 30 min on ice, the lysates were clarified by centrifugation and applied to a 1 ml HisTrap™ column or purified in batch. pH of all buffers was non-standard because lysis and purifications at pH > 6.2 rendered His_6_-TAT-BoNT/B-LC insoluble a few hours after elution. The high theoretical isoelectric point of His_6_-TAT-BoNT/B-LC (pI = 7.9) explains its insolubility behavior at neutral and alkaline pHs (not shown). Untagged versions of the toxin have lower pI (His_6_-BoNT/B-LC = 6.22 and BoNT/B-LC = 5.9, http://isoelectric.org/) and do not precipitate at neutral pH.

The resin was washed with lysis buffer containing 20 mM imidazole before eluting the toxin with 250 mM imidazole. Aliquots of collected fractions were electrophoresed on 10% (w/v) SDS gels; expression and purification were assessed by Coomassie Brilliant Blue R250 staining. His_6_-TAT-BoNT/B-LC was highly expressed in *E. coli* and purified to near homogeneity by Ni^2+^ affinity chromatography (Figure 1B). Eluates enriched in the toxin were pooled, diluted two fold in lysis buffer without imidazole, and added glycerol. Protein concentration was determined by the Bradford method (Bio-Rad) using bovine serum albumin (BSA) as a standard in 96-well microplates and quantified on a BioRad 3550 Microplate Reader. The recombinant protein in storage buffer (50 mM NaH_2_PO_4_, pH 6.2, 300 mM NaCl, 125 mM imidazole, 10% (v/v) glycerol), aliquoted and stored at −20°C, remained soluble and active for months. The highest protein concentration achieved was ≈ 28 μM before dilution, and total recovery was ≈ 3.75 mg from a 250 ml culture.

### Expression and purification of His_6_-BoNT/B-LC

The light chain of BoNT/B fused to His_6_ in a pQE3 vector was generously provided by Dr. T. Binz (Medizinische Hochschule Hannover, Hannover, Germany). Expression and purification of recombinant His_6_-BoNT/B-LC were as in [13].

### His_6_-TAT-BoNT/B-LC delivery to synaptosomes

Rat brain synaptosomes were incubated for 30 min at 37°C with His_6_-TAT-BoNT/B-LC or His_6_-BoNT/B-LC in 20 mM Hepes-NaOH, pH 7.8, 100 mM KCl, 1% (v/v) glycerol, 1 mM DTT, 2.5 mM ATP, and 2 mM MgCl_2_. Uptake and enzymatic activity were assessed by anti-synaptobrevin-2 Western blot. Anti-syntaxin1 was used as loading control.

### Sensitivity of His_6_-TAT-BoNT/B-LC to trypsin

One microgram of purified His_6_-TAT-BoNT/B-LC was incubated with 0.05–1.5 ng trypsin in 15 μl HTF. After 20 min at 37°C, samples were boiled, aliquoted, loaded on 10% (w/v) SDS-polyacrylamide gels and electrophoresed. Half the gels were stained with Coomassie Brilliant Blue R250 and the other half transferred to nitrocellulose and processed for Western blot with anti-His_6_ antibodies.

### Trypsin protection assay for the His_6_-TAT-BoNT/B-LC translocated into human sperm cells

Human sperm cells sample preparation procedures (isolation of motile cells from semen, capacitation, permeabilization of the plasma membrane with streptolysin O (SLO) and indirect AR assays in permeabilized and non-permeabilized sperm cells were performed as described by Bustos et al [14]. Sperm cells incubated under capacitating conditions were washed and resuspended in HTF without BSA (30 × 10^6^ cells in 300 μl) and incubated with 1 μM His_6_-TAT-BoNT/B-LC for 30 min at 37°C. After washing once with PBS, sperm cells were exposed to 0.1 μg/ml trypsin (Sigma) for 20 min at 37°C. Following centrifugation, proteins from the supernatants were boiled in reducing sample buffer. Sperm cells were washed once with PBS, boiled for 3 min at 95°C in 50 μl of non-reducing sample buffer. Extracted proteins were clarified by centrifugation, added beta mercaptoethanol, boiled as before and analyzed by Western blot with anti-His_6_ antibodies to detect the internalized recombinant protein.

### Synaptobrevin sensitivity to His_6_-TAT-BoNT/B-LC translocated into human sperm cells

Capacitated sperm cells suspensions (10^7^/ml in HTF) were incubated with 1 μM His_6_-TAT-BoNT/B-LC or His_6_-BoNT/B-LC 30 min at 37°C before challenging with 10 μM A23187. Acrosomal and nuclear staining and synaptobrevin-2 immunofluorescence were carried out as in [15,16].

### SDS-PAGE and Western blot

Proteins were resolved by electrophoresis on SDS-gels and electro-transferred to 0.22 μm nitrocellulose membranes (Hybond, GE HealthCare) on a semi-dry apparatus (Amersham Biosciences) for 80 min at 25 mA. Non-specific reactivity was blocked with 2% (w/v) BSA dissolved in washing buffer (PBS, pH 7.6, 0.1% (v/v) Tween 20) for 1 h at room temperature. Blots were incubated with 5 ng/ml anti-His_6_, 20 μg/ml anti-synaptobrevin-2 or 1:2000 anti-syntaxin antibodies in blocking solution overnight at 4°C. Horseradish peroxidase-conjugated goat-anti-mouse IgG (0.1 μg/ml in washing buffer) was used as secondary antibody with 1 h incubation at room temperature. Excess first and second antibodies were removed by rocking in washing buffer three times, 10 min each. Detection was accomplished with a chemiluminescence kit from Kalium Technologies (Biolumina, Buenos Aires, Argentina) on a Luminescent Image Analyzer LAS-4000 (Fujifilm, Tokyo, Japan). Full uncropped versions of Western blots are shown in Supplementary Figure S1. Quantification of signal intensities was carried out with Image Studio™ Software Lite 5.2 (LICOR Bio™).

### Ethics

The research has been carried out in accordance with the World Medical Association Declaration of Helsinki. Human subjects were involved in this project for the purpose of semen donation. The subject population consisted of healthy male donors 21 years of age or over. All subjects signed a written informed consent form at the time of their enrollment. The Bioethical Committee of the Medical School (Comité de Bioética de la Facultad de Ciencias Médicas de la Universidad Nacional de Cuyo) approved our protocol for the collection and manipulation of human sperm cells samples (University's equivalent to IRB number 5892/2020). All laboratory procedures followed the safety regulations of the Medical School.

## Results and discussion

### Design and purification of His_6_-TAT-BoNT/B-LC

The map of the plasmid encoding the cell penetrating toxin is delineated in Figure 1A. His-tags potentially influence both protein oligomeric states and function [17,18]. To gain insights into the impact of the His_6_-TAT peptide on the folding behavior of recombinant His_6_-TAT-BoNT/B-LC, we computationally modeled its structure using RoseTTAfold and i-Tasser based on its amino acid sequence [19,20]. As depicted in Figure 1C,D, the three-dimensional structure of the protein predicted by RoseTTAfold, closely resembled the crystal structure of the BoNT/B light chain (PDB: 2ETF, RMSD = 0.94 for 350 Cα). Experimental data reported in the literature suggest the TAT peptide adopts an unstructured conformation [21]. Most models of His_6_-TAT-BoNT/B-LC generated by RoseTTAfold and i-Tasser also predicted an unstructured TAT (data not shown). Even when considering a model where the peptide folded into an alpha helix (Figure 1C,D, blue), no interactions with, or hindrances to, the catalytic site (Figure 1C,D, pink) were predicted. Thus, we infer that the TAT peptide is exposed in His_6_-TAT-BoNT/B-LC, a desirable feature for binding membranes and transducing cargo, and that it will not interfere with the enzymatic activity of the toxin.

### His_6_-TAT-BoNT/B-LC permeates through synaptosomal membranes and cleaves synaptobrevin-2

BoNT/B specifically cleaves the integral SNARE protein synaptobrevin at the peptide bond Q76-F77 located in the SNARE motif (residues 30-85) in the cytoplasmic region of the protein [22]. Synaptosomes are synaptic terminals isolated from neurons surrounded by resealed plasma membrane. Synaptosomes from rat cerebral cortex contain synaptobrevin-2 bound to the synaptic vesicles in their interior.

We used synaptosomes as a test system for the membrane permeating and proteolytic activity of the His_6_-TAT-BoNT/B-LC. We reasoned that if the toxin penetrated through the synaptosomal membrane, it would reach its substrate synaptobrevin-2 and cleave it. To distinguish between intact and proteolyzed synaptobrevin, we used the monoclonal antibody 69.1, raised against the 17 residues at the amino terminus end of synaptobrevin-2 [23]. Clone 69.1 binds with high affinity to synaptobrevin-2, monomeric and assembled in ternary SNARE complexes but it does not detect synaptobrevin cleaved by BoNT/B even though the severed cytosolic fragment contains the 17 amino terminus residues. Likewise, an unrelated rabbit polyclonal antibody raised against the 20 residues at the amino-terminal of synaptobrevin-2 does not detect the 12 kDa fragment produced by BoNT/B cleavage [24].

**Figure 2:**
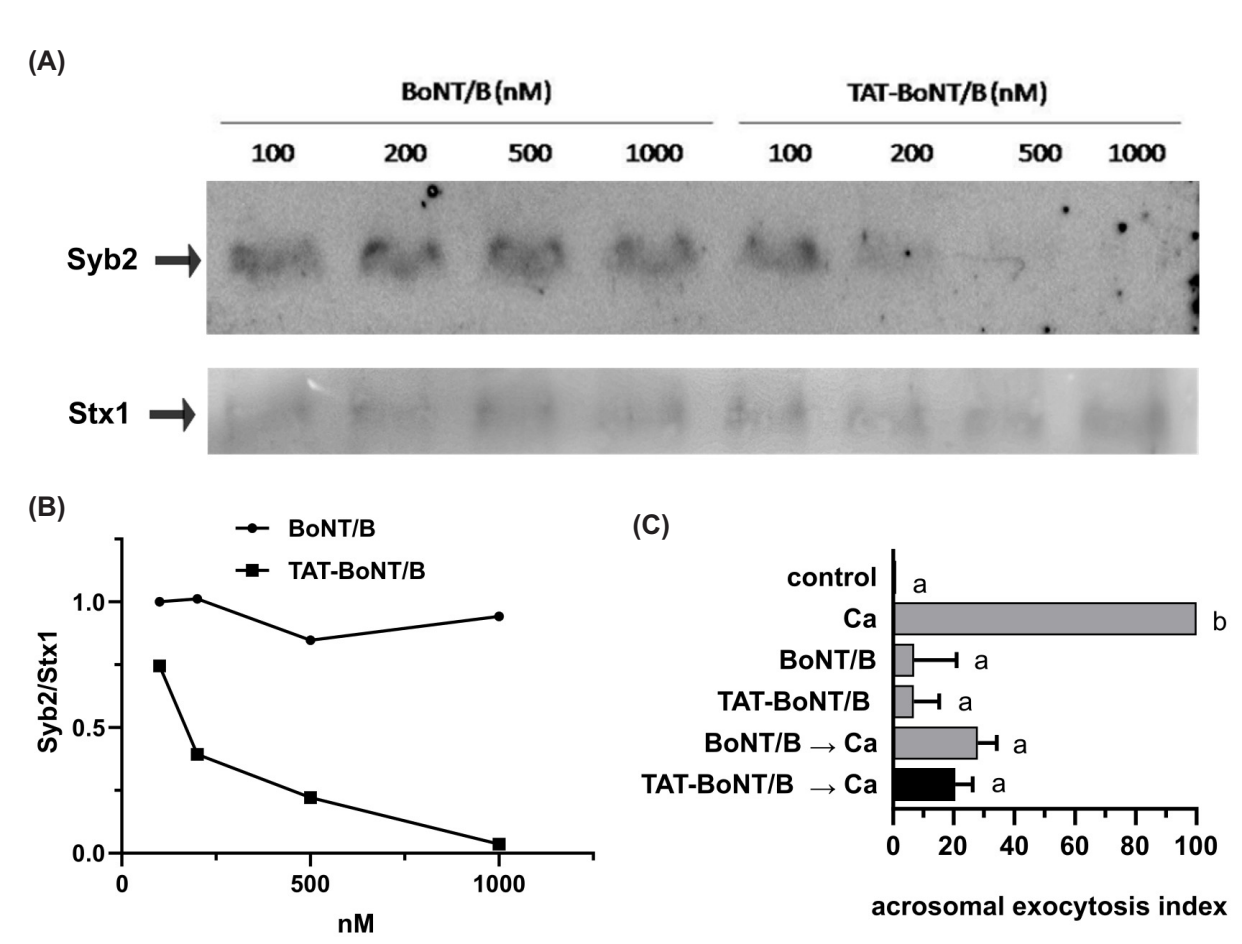
His_6_-TAT-BoNT/B-LC crosses synaptosomal membranes and is enzymatically active (**A**) A rat brain preparation enriched in synaptosomes was incubated for 30 min at 37°C with increasing concentrations of His_6_-BoNT/B-LC (left) of His_6_-TAT-BoNT/B-LC (right) as described in the Methods section. Reactions were terminated by addition of SDS sample buffer and the amount of intact synaptobrevin-2 was analyzed by Western blot using the 69.1 antibody as probe. The arrow indicates the electrophoretic mobility of synaptobrevin-2 (Syb). Anti-syntaxin 1 (Stx1) blot shows equal loading of synaptosomal proteins. Shown are blots representative of three repetitions. (**B**) Quantification was expressed as the ratio of the signal intensities of synaptobrevin-2 relative to syntaxin1 when synaptosomes were incubated with His_6_-TAT-BoNT/B-LC (squares) or His_6_-BoNT/B-LC (circles). (**C**) 100 nM His_6_-BoNT/B-LC and His_6_-TAT-BoNT/B-LC (black bar) were introduced into SLO-permeabilized sperm cells by incubating at 37°C for 15 min. The AR was induced with 0.5 mM CaCl_2_ and incubating as before. Sperm cells were fixed and the AR was measured by FITC-PSA binding as described in [14]. Gray bars represent controls: background (control), AR stimulated by 0.5 mM CaCl_2_ (Ca), lack of effect of the toxins on the basal AR (BoNT/B, TAT-BoNT/B) and inhibitory effect of the not-membrane penetrating version on the AR elicited by CaCl_2_. The data represent the mean ± SEM of at least three independent experiments. Data were evaluated before normalization with the program GraphPad Prism 8 using the one way Anova, Dunnett's multiple comparisons test. Different letters indicate statistical significance (*P*<0.05).

We incubated synaptosomes with 100, 200, 500 and 1000 nM His_6_-TAT-BoNT/B-LC and evaluated the integrity of synaptobrevin-2 by Western blot with the monoclonal antibody 69.1 (Syb2). The signal substantially decreased when synaptosomes were treated with 500 nM His_6_-TAT-BoNT/B-LC and disappeared with 1000 nM toxin (Figure 2A, top and Figure 2B, squares). These results suggest that His_6_-TAT-BoNT/B-LC penetrated through the synaptosomes’ membranes within minutes and attacked synaptobrevin. When we applied His_6_-BoNT/B-LC, synaptobrevin remained uncut even at 1000 nM toxin (Figure 2A, top and Figure 2B, circles). Synaptosomal syntaxin1 (Stx1) was used as loading control. We conducted the next series of experiments to reassure that the inability of His_6_-BoNT/B-LC to cleave synaptosomal synaptobrevin-2 was due to lack of permeability and not of activity.

Sperm cells undergo regulated exocytosis in response to calcium; secretion relies on the same exocytotic machinery as in somatic cells, including toxin-sensitive SNARE proteins [25]. Thus, introducing His_6_-BoNT/B-LC into human sperm cells with their plasma membrane permeabilized with SLO cleaves synaptobrevin and inhibits exocytosis [13]. His_6_-TAT-BoNT/B-LC introduced into SLO-permeabilized sperm cells hindered the AR elicited by calcium as did His_6_-BoNT/B-LC (Figure 2C). As expected, neither toxin had any effect on basal exocytosis. These results indicate that both versions of BoNT/B were enzymatically active. They also confirm that His_6_-BoNT/B-LC failed to cut synaptosomal synaptobrevin because the toxin and its substrate were in different compartments.

### His_6_-TAT-BoNT/B-LC translocates into human sperm cells and inhibits exocytosis

**Figure 3:**
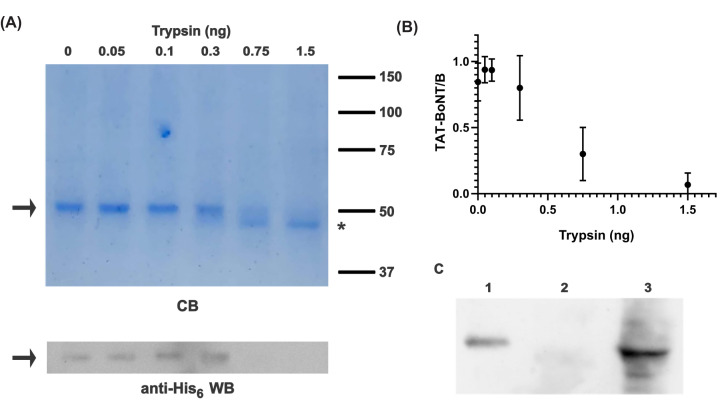
His_6_-TAT-BoNT/B-LC penetrates into human sperm cells (**A**) 1 μg purified His_6_-TAT-BoNT/B-LC was incubated with increasing amounts of trypsin as indicated in the Methods section. Top: Coomassie Brilliant Blue R250 (CB) stained gel; 2/3 of each sample were run per lane. The full length protein (arrow) disappeared and a band with higher mobility (asterisk) appeared with the higher amounts of trypsin tested. *Mr* standards (×10^3^ Da) are indicated on the right. Bottom: aliquots of the same samples were subjected to anti-His_6_ Western blot; 1/3 of each sample were run per lane. The antibodies detected full length His_6_-TAT-BoNT/B-LC (arrow). Shown is an experiment representative of three repetitions. (**B**) Quantification of the anti-His_6_ signal in uncleaved His_6_-TAT-BoNT/B-LC depicted as mean ± SD from all replicates. (**C**) Capacitated human sperm cells were incubated with 1 μM His_6_-TAT-BoNT/B-LC, washed and treated with 0.1 μg/ml trypsin as indicated in the Methods section. Samples were centrifuged and proteins from both the sperm cells pellet (incorporated, lane 3) and the extracellular supernatant (unincorporated, lane 2) were processed for anti-His_6_ Western blot. Lane 1 shows His_6_-TAT-BoNT/B-LC equivalent to that found in the supernatant of mock experiments conducted without trypsin (1 µg). Shown is an experiment representative of four repetitions.

Sperm cells are an ideal model system to study the delivery of membrane penetrating proteins because they do not undergo endocytosis. Hence, a protein added to the medium and found inside the cell has inevitably entered through transduction. Sensitivity to trypsin allows us to distinguish between proteins bound to the surface of the cells – sensitive to trypsin – and those incorporated into the cells - resistant to trypsin. Figure 3A illustrates *in vitro* experiments where His_6_-TAT-BoNT/B-LC exposed to increasing concentrations of trypsin was subsequently electrophoresed and stained with Coomassie Brilliant Blue R250 (A, top) or transferred to nitrocellulose and probed with anti-His_6_ antibodies by Western blot (A, bottom, quantification is shown in B). At 4.3 nM trypsin, the band of intact His_6_-TAT-BoNT/B-LC (arrow) disappeared and a smaller, faster band (asterisk) appeared to its expense in the Coomassie-stained gel. The smaller band was not observed in the Western blot, which indicates that trypsin had removed its His_6_ tag. Once we had established the minimal concentration of trypsin rendering His_6_-TAT-BoNT/B-LC undetectable by the antibody, we incubated human sperm cells with the toxin, added trypsin to remove the excess as well as that bound to the surface, and analyzed the results by Western blot with anti-His_6_ antibodies. Figure 3C shows that His_6_-TAT-BoNT/B-LC translocated into sperm cells rapidly and efficiently (20% of the input translocated in 30 minutes). This kinetics is much faster than that of native BoNT/B, the internalization of which via endocytosis and translocation of the LC to the cytoplasm of somatic cells take hours, or even days [8,26–29]. Figure 3C also shows that trypsin erased the His_6_ signal from the remaining His_6_-TAT-BoNT/B-LC in the supernatant after recovering the cells by centrifugation. These results indicate that the amount of trypsin used was sufficient to proteolyze any toxin that may have attached to the outer leaflet of the sperm plasma membrane.

Our group has broad experience in translocating proteins coupled to CPPs into human sperm cells, for example GST [30], Rab27A [14], a cyclic AMP sponge [31] and metallothionein [32]. In all these cases, delivery was attested by insensitivity to trypsin and revealed with anti-tag antibodies by immunofluorescence and/or Western blot.

**Figure 4:**
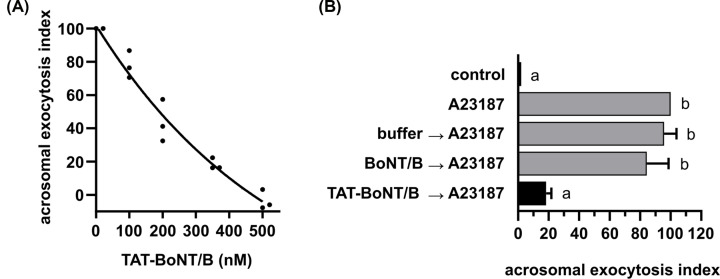
His_6_-TAT-BoNT/B-LC translocated into human sperm cells inhibits the AR (**A**) Capacitated human sperm cells were exposed to increasing concentrations of His_6_-TAT-BoNT/B-LC for 15 min at 37°C. Acrosomal exocytosis was initiated with 10 µM A23187 and incubating as before. (**B**) 350 nM His_6_-BoNT/B-LC and His_6_-TAT-BoNT/B-LC (black bar) were introduced into capacitated sperm cells incubating at 37°C for 15 min. The AR was induced with 10 µM A23187 and incubating as before. Sperm cells were fixed and the AR was measured by FITC-PSA binding as described in [14]. Gray bars represent controls: background (control), AR stimulated by 10 µM A23187 (A23187), lack of effect of the elution buffer and of the not-membrane penetrating version on the AR elicited by A23187. The data represent the mean ± SEM of at least three independent experiments. Data were evaluated before normalization with the program GraphPad Prism 8 using the one-way Anova, Dunnett’s multiple comparisons test. Different letters indicate statistical significance (*P*<0.05).

Next, we tested the effect of His_6_-TAT-BoNT/B-LC on the AR triggered by the calcium ionophore A23187. The toxin inhibited exocytosis in a dose–response manner, with 350 nM His_6_-TAT-BoNT/B-LC inhibiting the AR by ≈ 80% and 500 nM preventing it completely (Figure 4A). His_6_-BoNT/B-LC applied at the same concentration as His_6_-TAT-BoNT/B-LC (350 nM, Figure 4B) did not inhibit the AR. The difference in the amount of toxin required to inhibit by 80% the AR triggered by calcium in SLO-permeabilized sperm cells (100 nM, Figure 2B) and by A23187 in non-permeabilized (350 nM, Figure 4A) was small. In the former, His_6_-TAT-BoNT/B-LC had free access to the interior of the cells whereas in the latter it needed to gain access permeating through the plasma membrane. These findings reinforce the notion that the TAT peptide was able to translocate its cargo across the plasma membrane and deliver it into the cytoplasm efficiently and quantitatively. A membrane penetrating version of TeTx-LC exhibits lower efficiency or potency: 1.5 μM applied for 15 min inhibited the AR elicited by A23187 or ceramide-1-phosphate by ≈ 50% [33,34] and 1 μM applied for 30 min, by 40% [12]. Furthermore, the His_6_-TAT-BoNT/B-LC described herein has the advantage of being bacterially expressed as a single protein fused to the CPP. The time and effective concentration of His_6_-TAT-BoNT/B-LC described herein are comparable to those required by TAT-BoNT/A-LC to permeate into cultured HeLa and neuronal [BE(2)-C] cell lines, and mouse skin (3 μM, 30 min) [11].

### His_6_-TAT-BoNT/B-LC permeates through sperm cells membranes and cleaves synaptobrevin-2

**Figure 5:**
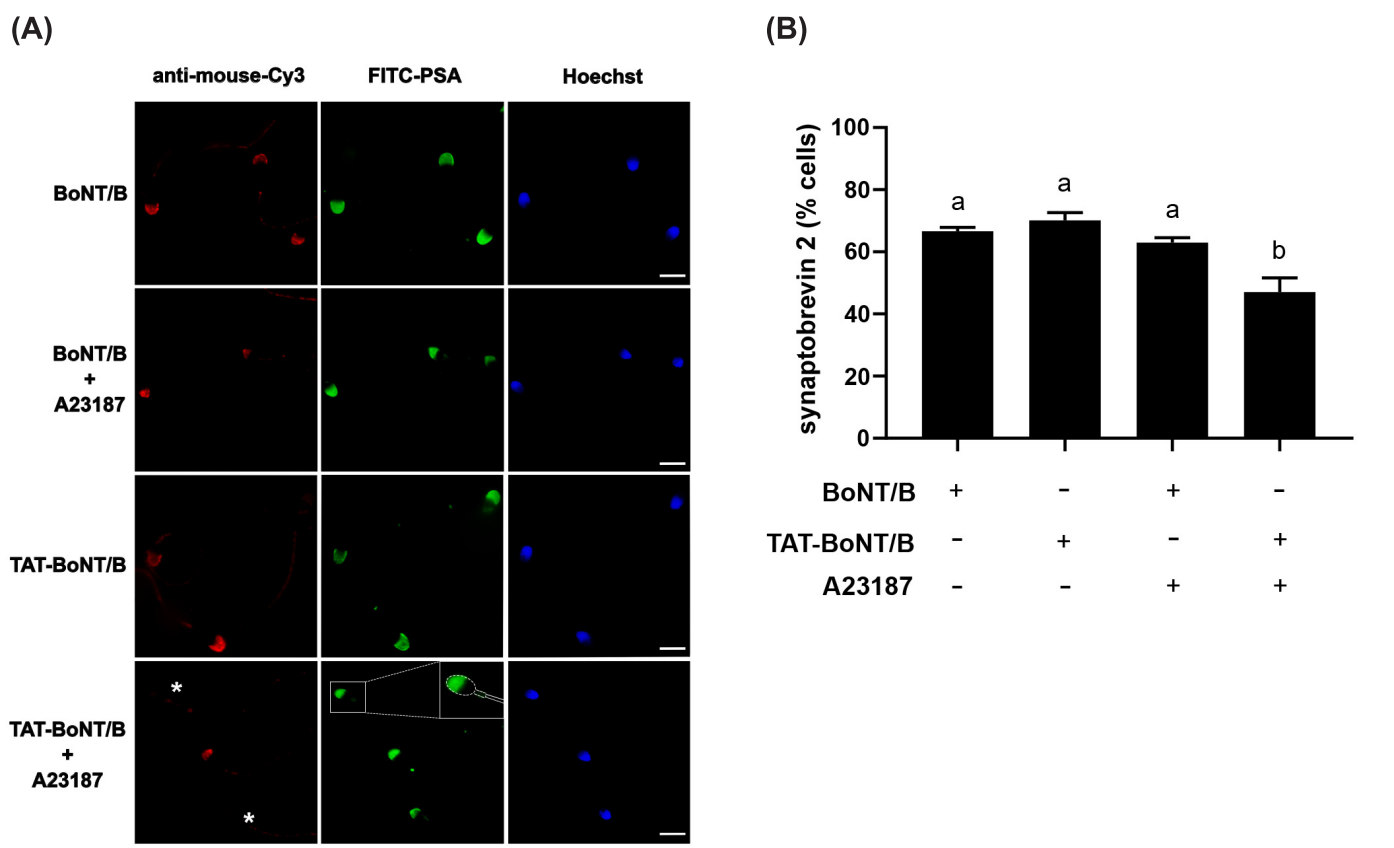
His_6_-TAT-BoNT/B-LC translocated into human sperm cells cleaves synaptobrevin (**A**) Sperm cells incubated as indicated in the figure key were fixed and triple stained with an anti-synaptobrevin-2 antibody followed by a fluorescent secondary antibody (anti-mouse-Cy3, red, left panels), FITC-PSA (green, central panels), and Hoechst 33342 (blue, right panels). Samples were examined with an 80i Nikon microscope equipped with a Plan Apo 60x/1.40 oil objective. All images were captured - with the same length of exposure for the acquisitions - with a Nikon DS-Fi1 camera operated with NIS software (Nikon). ImageJ (freeware from N.I.H.) was used to subtract background and adjust brightness/contrast to render all-or nothing labeling patterns. The presence of immunostaining in the acrosomal region was scored in ≈ 20 digital images from at least ≈200 cells. Shown are representative images of sperm cells with intact acrosomes and synaptobrevin-2 staining and without synaptobrevin-2 immunostaining due to toxin cleavage (asterisks). Bars = 5 μm. The silhouette of the head of a sperm cell is delineated with dashed bars (bottom, green). (**B**) Quantification of the percentage of non-exocytosing sperm cells with synaptobrevin-2 staining. The data represent the mean ± SEM of three independent experiments: BoNT/B: 208-201-206 cells counted; BoNT/B+A23187: 227-222-210 cells counted; TAT-BoNT/B: 205-232-218 cells counted; TAT-BoNT/B+A23187: 230-254-223 cells counted. Data were evaluated with the program GraphPad Prism 8 using the one-way Anova, Dunnett’s test multiple comparisons test. Different letters indicate statistical significance (P<0.05).

We tested the premise that His_6_-TAT-BoNT/B-LC inhibited sperm cells exocytosis because it digested synaptobrevin-2 by probing its integrity with the monoclonal antibody 69.1 in immunofluorescence experiments. We scored immunostaining in unreacted cells because sperm that undergo exocytosis lose the membranes containing synaptobrevin; hence, lack of staining in reacted cells cannot unequivocally be attributed to SNARE cleavage. We had previously established that SNARE proteins are engaged in toxin-insensitive *cis* complexes in capacitated cells. When human sperm cells were exposed to His_6_-TAT-BoNT/B-LC and tripled stained for intact synaptobrevin (69.1 as primary and anti-mouse-Cy3 IgGs as secondary antibodies, red, left panels), acrosome (labeled with FITC-PSA, green, central panels) and DNA (nuclei, blue, right panels), 70% of unreacted (green) cells were decorated by the antibody (Figure 5 ‘TAT-BoNT/B’). The anti-synaptobrevin-2 antibody stained the acrosomal region despite pretreatment with His_6_-TAT-BoNT/B-LC, corroborating that SNAREs were in a *cis* configuration in resting sperm cells.

Once inducers initiate the AR, complexes disassemble, and SNAREs become susceptible to toxin cleavage (reviewed in [25]). Approximately 47% of unreacted cells showed intact synaptobrevin when sperm cells were exposed to the toxin and A23187 (Figure 5 ‘TAT-BoNT/B + A23187’). The proportion of cells exhibiting synaptobrevin-2 labeling dropped significantly because the initiation of the AR sensitized this SNARE to BoNT/B. In contrast, the percentages of unreacted cells with red staining were similar regardless of the presence or absence of A23187 when the not-penetrating version of the toxin was used (Figure 5 ‘BoNT/B’ vs ‘BoNT/B + A23187’). These results indicate that His_6_-TAT-BoNT/B-LC penetrated through the plasma membrane and cleaved synaptobrevin-2 when sperm cells were challenged to undergo exocytosis. Such proteolysis explains the inhibition of the AR by the toxin (Figure 4). Likewise, the integrity of synaptobrevin-2 in sperm cells exposed to the not-membrane penetrating version of the toxin was consistent with its failure to inhibit the AR (Figure 4B).

The novel findings reported here include the description of membrane-penetrating His_6_-TAT-BoNT/B-LC as a trustworthy tool to unveil the involvement of synaptobrevin in sperm cells exocytosis under physiological conditions; this tool may be applied to any secretory cell. In the long term, it may eventually be used to optimize therapeutic utilization of BoNT/B and/or to contribute to the development of counteragents against malicious toxin applications. Our acute delivery approach confers an advantage over methods that genetically modify their targets because the cells, their machinery, maturation and properties are preserved. Furthermore, His_6_-TAT-BoNT/B-LC can be used in cells unable to synthesize proteins. Finally, His_6_-TAT-BoNT/B-LC is non-toxic and can be handled in the laboratory without the high risks involved in working with the holotoxin.

## Supplementary Material

Supplementary Material S1 and Figure S1

## Data Availability

Data that support the findings of this study and associated protocols will be made available upon request.

## Abbreviations

AR: acrosome reaction
BoNT/B: botulinum toxin B
BSA: bovine serum albumin
CPP: cell penetrating peptide
FITC: fluorescein isothiocyanate
HTF: human tubal fluid
IPTG: isopropyl-β-D-thio-galactoside
LC: light chain
PBS: phosphate buffer saline
PSA: *Pisum sativum* agglutinin
SDS-PAGE: sodium dodecyl sulfate-polyacrylamide gel electrophoresis
SLO: streptolysin O
SNARE: SNAP receptor
TeTx: tetanus toxin
