# Supplementary material for: Expression, purification and application of a recombinant, membrane permeating version of the light chain of botulinum toxin B
Source: Biosci Rep. 2024 Jul 30;44(7):BSR20240117. doi: 10.1042/BSR20240117 (PMC11292472; doi:10.1042/BSR20240117)
Supplement: Supplementary Material S1 and Figure S1 [file BSR-2024-0117_supp.zip › BSR-2024-0117_suppSM1.docx]

**Supplementary Material 1**

**Optimized Sequence** (Optimized Sequence Length:1320, GC%:48.38)

CCGGTGACCATTAACAACTTTAACTACAACGACCCGATCGATAACAACAACATCATTATGATGGAGCCGCCGTTT

GCGCGTGGCACCGGCCGTTACTATAAAGCGTTCAAGATCACCGACCGTATTTGGATCATTCCGGAGCGTTACACC

TTCGGTTATAAACCGGAAGACTTTAACAAGAGCAGCGGCATCTTCAACCGTGATGTGTGCGAATACTATGACCCG

GATTACCTGAACACCAACGACAAGAAAAACATCTTTCTGCAGACCATGATTAAACTGTTCAACCGTATCAAGAGC

AAACCGCTGGGCGAGAAGCTGCTGGAAATGATCATTAACGGTATCCCGTATCTGGGCGATCGTCGTGTTCCGCTG

GAGGAGTTCAACACCAACATTGCGAGCGTGACCGTTAACAAACTGATCAGCAACCCGGGTGAGGTGGAGCGTAAG

AAAGGCATTTTCGCGAACCTGATCATTTTTGGTCCGGGCCCGGTTCTGAACGAGAACGAAACCATCGACATTGGT

ATCCAGAACCACTTCGCGAGCCGTGAGGGCTTTGGTGGCATCATGCAAATGAAATTTTGCCCGGAGTACGTGAGC

GTTTTCAACAACGTGCAAGAAAACAAGGGTGCGAGCATTTTCAACCGTCGTGGCTACTTTAGCGATCCGGCGCTG

ATTCTGATGCACGAGCTGATCCACGTTCTGCACGGTCTGTATGGCATTAAGGTGGACGATCTGCCGATCGTTCCG

AACGAAAAGAAATTCTTTATGCAGAGCACCGACGCGATCCAAGCGGAGGAACTGTACACCTTTGGTGGCCAGGAC

CCGAGCATCATTACCCCGAGCACCGACAAAAGCATTTATGATAAGGTGCTGCAAAACTTCCGTGGTATCGTTGAC

CGTCTGAACAAAGTGCTGGTTTGCATTAGCGATCCGAACATCAACATCAACATCTACAAGAACAAGTTCAAGGAC

AAGTACAAGTTTGTGGAGGATAGCGAGGGTAAGTACAGCATCGACGTTGAGAGCTTCGATAAACTGTATAAGAGC

CTGATGTTCGGCTTTACCGAGACCAACATTGCGGAAAACTACAAAATCAAGACCCGTGCGAGCTATTTTAGCGAC

AGCCTGCCGCCGGTTAAAATCAAGAACCTGCTGGATAACGAAATCTACACCATTGAGGAAGGTTTCAACATTAGC

GACAAAGATATGGAGAAGGAATATCGTGGCCAGAACAAAGCGATTAACAAGCAAGCGTACGAGGAAATCAGCAAA

GAACACCTGGCGGTGTATAAGATCCAGATGTGCAAAAGCGTTAAG

**Supplementary Material 1:** Nucleotide sequence of cDNA encoding His_6_-TAT-BoNT/B-LC.
